# Transformation from a Single Antenna to a Series Array Using Push/Pull Origami

**DOI:** 10.3390/s17091968

**Published:** 2017-08-26

**Authors:** Syed Imran Hussain Shah, Sungjoon Lim

**Affiliations:** School of Electrical and Electronics Engineering, College of Engineering, Chung-Ang University, 84 Heukseok-ro, Dongjak-gu, Seoul 156-756, Korea; engr.shahsyedimran@gmail.com

**Keywords:** push/pull origami, three-dimensional (3D) printing technology, inkjet printing technology, hybrid printing technology

## Abstract

We propose a push/pull origami antenna, transformable between a single antenna element and a three-element array. In limited space, the proposed origami antenna can work as a single antenna. When the space is not limited and a higher gain is required, the proposed origami antenna can be transformed to a series antenna array by pulling the frame. In order to push the antenna array back to a single antenna, the frame for each antenna element size must be different. The frame and supporting dielectric materials are built using a three-dimensional (3D) printer. The conductive patterns are inkjet-printed on paper. Thus, the proposed origami antenna is built using hybrid printing technology. The 10-dB impedance bandwidth is 2.5–2.65 GHz and 2.48–2.62 GHz for the single-antenna and array mode, respectively, and the peak gains in the single-antenna and array mode are 5.8 dBi and 7.6 dBi, respectively. The proposed antenna can be used for wireless remote-sensing applications.

## 1. Introduction

Origami folding enables one to transform paper into various shapes and configurations, without using any adhesives or cuts. This art is expanded by exploring various materials, cuts, and joints. Origami has attracted attention because of its advantages of deployability, portability, easy manufacturing procedure (folding), ease of miniaturization, and low-cost materials (paper sheets) [1,2]. Engineers and scientists are exploring origami applications in various fields such as architecture, energy harvesting, and spacecraft applications. The origami features are attractive for antenna design applications, where high portability and deployability are often required. Deployable origami antennas can also be used in space applications [3,4,5]. For instance, a deployable origami helical antenna can be used for nanosatellites such as CubeSat [3]. In [4,5], frequency-reconfigurable spring-shaped antennas were proposed for satellite applications. In addition, nano-sized origami can be used for medical applications [6,7]. A nano-sized origami spring was designed to serve as programmable DNA [6]. In [7], nano-sized origami device was used in self-deployable stent grafts for medical applications. In [8], an origami concept was used to design a high-gain tetrahedron-shaped antenna. A self-foldable microstrip patch antenna was presented in [9]. In [10], a mode-reconfigurable helical origami antenna was presented. In [11], a circularly polarized origami antenna was designed for military field deployment.

In addition, 3D printing technology is an additive manufacturing (AM) process used for fabricating 3D objects. In this process, successive layers of dielectric materials are printed under computer control. In comparison to conventional metal etching, AM processes have shown several advantages such as lower fabrication cost, easy and fast printing, and efficient material utilization. Various complex designs can be printed by using 3D printing technology, which are not possible with conventional etching processes. Therefore, in literature, some antenna and radio-frequency (RF) circuits have been presented by using 3D printing technology. For instance, in [12], a circularly polarized patch antenna was fabricated by using 3D printing technology. In [13], an efficient microstrip patch antenna was designed using meshed poly-lactic acid (PLA) by a 3D printer. Since conductive patterns are necessary for antennas and RF circuits, numerous metal printing methods for 3D-printed antennas have been presented in literature. For instance, an ultrasonic wire-mesh embedding method was used for printing the conductive pattern in [14]. However, handling an ultrasonic welding machine can be hazardous because of its high voltage and heat level, which can also damage the 3D-printed substrate [15]. The metallic patterns of the antenna can be printed by using an inkjet printer. Compared to conventional metal etching [16,17], this conductive printing process is fast and inexpensive, and suitable for printing planar metallic patterns such as microstrip patch antennas. In [18], nanocomposite ink was presented and characterized for RF applications. The proposed nanocomposite ink can reduce the required sintering temperature and achieve fine printing resolution. In [19], the microstrip lines were inkjet-printed on an alumina substrate, which takes advantage of the fast prototyping for high-frequency applications. In [20], a low-cost inkjet-printed microstrip patch antenna was realized on an FR4 substrate.

Therefore, considering the advantages of origami and hybrid printing technology, we designed a push/pull origami antenna by using hybrid-printing technology. The proposed antenna can be transformed to either a single antenna element or a series 3 × 1 antenna array, depending on the availability of space for the antenna and the gain requirement. Three antenna elements were fabricated with different frame sizes. In limited space, for single-antenna-element operation, the third antenna-element frame can be pushed within the frame of the second antenna element, which is pushed beneath the first antenna element. When the space is not limited and a higher gain is required, the proposed origami antenna can be transformed into a 3 × 1 series array by pulling out the second and third antenna elements. In this method, the second and third antenna elements are aligned with the first antenna element, and the antenna works in the array mode. Hence, the push/pull origami mechanism is a key feature of the proposed antenna. The second and third array elements are series-fed with a small gap in the feeding line. The antenna was fabricated using hybrid printing technology. The frame and supporting dielectric material parts were built using a 3D printer. The conductive patterns were inkjet-printed on paper. Compared to previously reported origami antennas [1,2,3,4,5,8,9,10,11], the proposed origami antenna is robust because it is built on 3D-printed PLA. The 10-dB impedance bandwidths for the single-antenna mode and array mode are 2.5–2.65 GHz and 2.48–2.62 GHz, respectively. The peak gains of the single antenna and array are 5.8 dBi and 7.6 dBi, respectively.

## 2. Antenna Design

We first designed a microstrip patch antenna to resonate at 2.6 GHz. The width (*W*) and length (*L*) of the rectangular patch are determined from well-known equations of microstrip patch antennas [21]:

(1) $$W=\frac{c\sqrt{\frac{2}{\varepsilon_{reff}+1}}}{2f_{0}}$$

(2) $$L=\frac{c}{2f_{0}\sqrt{\varepsilon_{reff}}}-2\Delta L$$

(3) $$\varepsilon_{reff}=\frac{\varepsilon_{r}{}_{}+1}{2}-\frac{\varepsilon_{r}-1}{2}{\left(1+12\frac{h}{w}\right)}^{-\frac{1}{2}}$$

**Figure 1:**
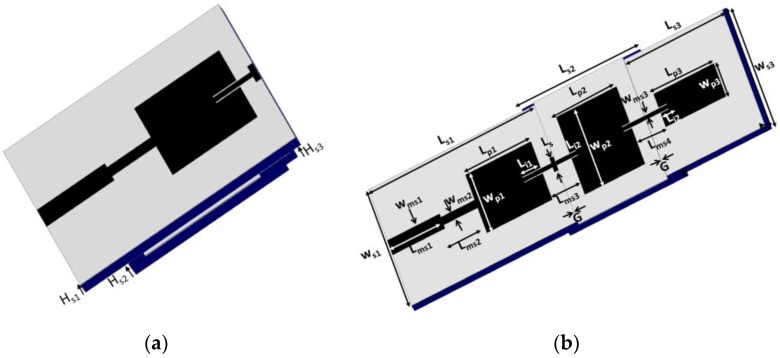
(**a**) Single antenna mode and (**b**) Three-antenna-element array mode.

**Figure 2:**
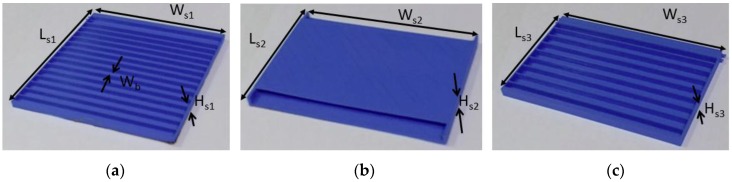
(**a**) 3D-printed PLA dielectric frame for the first antenna element, (**b**) 3D-printed hollow PLA frame for the second antenna element, and (**c**) 3D-printed PLA substrate frame for the third antenna element.

The length (L_P1_) and width (W_P1_) of the first patch are 50 mm and 60 mm, respectively. A quarter-wave transformer with length L_ms2_ and width W_ms2_ was designed for impedance matching. Then, the second and third antenna elements were designed with different frame sizes. The frame size of the second antenna element (Ls2 × Ws2 × Hs2) is larger than that of the third antenna element (Ls3 × Ws3 × Hs3). To ensure the stability of the paper on the 3D-printed frames, supporting beams with width W_b_ were built in the first and third antennas. The geometries of the antenna in the single antenna mode and array mode are shown in Figure 1. The frames of all the three elements are shown in Figure 2.

**Figure 3:**
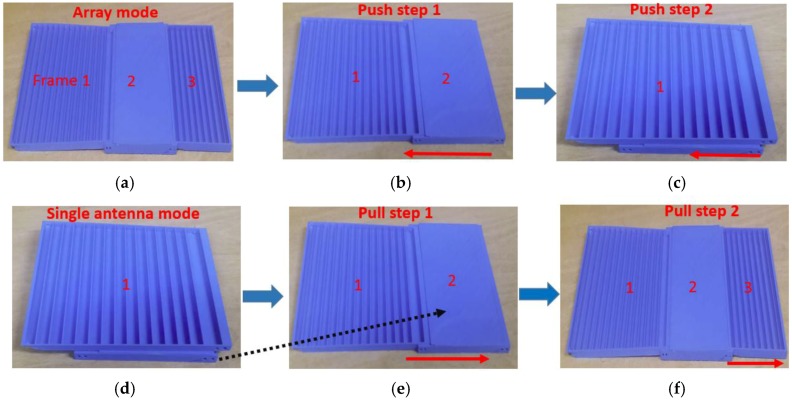
(**a**) Initial array mode, (**b**) Push the frame toward the single antenna mode, (**c**) Push the frame one more step to the single antenna mode, (**d**) Initial single antenna mode, (**e**) Pull the frame toward the array mode, (**f**) Pull the frame one more step to the array mode.

The frame of the second antenna element, as shown in Figure 2c, was printed hollow with four sidewalls, so that the third element could be accommodated in the second element. The push/pull origami mechanism can be understood from Figure 3. Initially, the proposed antenna works in the array mode, as shown in Figure 3a. To transform it into the single antenna, frame 3 is first pushed into frame 2, as shown in Figure 3b, and then frame 2 is pushed into frame 1, as shown in Figure 3c. The single antenna mode can be transformed back to the array mode. Figure 3d shows the initial state of the single antenna mode. To transform it into the array mode, frame 2 is first pulled out from frame 1, as shown in Figure 3e, and then frame 3 is pulled out from frame 2, as shown in Figure 3f. To maintain the same resonance frequency for the single-element and array modes, a tapered array was designed. The tapered array also has the advantage of a lower side-lobe level. The dimensions (length and width) of the patch of the second and third antenna elements are denoted by L_p2_/W_p2_ and L_p3_/W_P3_, respectively. All the patch elements are one wavelength apart. The length of the transmission line between the patch elements is equal to a half-wavelength to achieve the desired phase distribution of current on the radiating elements. An open-ended stub with length L_s_ was added in the series feed between the first and second antenna elements for better impedance matching in the array mode. The dimensions of all the antenna elements are given in Table 1.

## 3. Fabrication and Measurement Results

The proposed antenna was fabricated using 3D printing technology. The dielectric part of the antenna was built with a 3D printer. PLA was used as the 3D printer filament to fabricate the substrate of the antenna. PLA is a thermoplastic made from lactic acid. Compared to other thermoplastics, it has a much lower layer thickness and stronger bonding between layers [22]. In our previous work [13], PLA was characterized by designing a T-resonator. The dielectric constant and dielectric loss tangent of PLA were determined as 2.5 and 0.02, respectively. We used the PLA as the 3D printer filament because of its appreciable strength. Its diameter was 2.8 mm. The glass transition temperature and printing temperature of PLA are 55 °C and 180–230 °C, respectively. We used the Ultimaker 3D printer (Ultimaker B.V., Geldermalsen, The Netherlands), where the printing speed was set in the range of 40–100 mm/s. The diameter of the printer’s nozzle was 0.4 mm and it could print each layer with a thickness (Z-resolution) of 20 µm and line width (X/Y-resolution) of 400 µm. The minimum printable thickness depends on area of the printed wall. Although the minimum printable thickness is 0.3–0.4 mm, we printed minimum thickness of 0.5 mm, because of stability and repeatability. A Dimatix Materials Printer (DMP-2850, Fujifilm, Santa Clara, CA, USA) was used for printing the metallic pattern. DMC-11610 was used as the cartridge in the inkjet printer. The cartridge was filled with silver nanoparticle ink. Silver nanoparticle ink (JS-B25P, Novacentrix, Austin, TX, USA), which contains 25% silver, was used for the inkjet printing. Each droplet from the nozzle prints a spot of diameter 40 μm. To ensure uniform electrical conductivity, the pattern was printed thrice. Inkjet printing was performed on a photo paper. A Kodak photo paper having a thickness of 0.25 mm was used in this work. The paper was first characterized by designing a ring resonator. The dielectric constant and dielectric loss tangent of the paper were 2.1 and 0.02, respectively. The photo paper can withstand a temperature of 180 °C. Therefore, the conductivity of the silver nanoparticle ink can be improved with a sintering process. In this work, the inkjet-printed nanoparticle on paper was sintered at 150 °C for 20 min. The sintering process was performed in a drying oven (JEIO tech ON-22GW, Dongsung Science Co., Busan, Korea) before bonding the paper to 3D-printed PLA, because high temperature can damage the PLA. After the sintering process, the paper was bonded to the top side of the 3D-printed dielectric frames by using double-sided tape (31630-68338, Dongsung Science Co., Busan, Korea).

**Figure 4:**
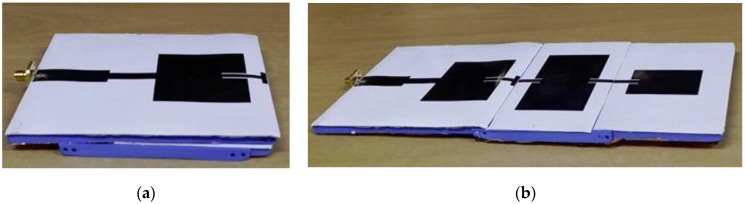
Fabricated prototype: (**a**) Single antenna mode and (**b**) Three-antenna-element array mode.

**Figure 5:**
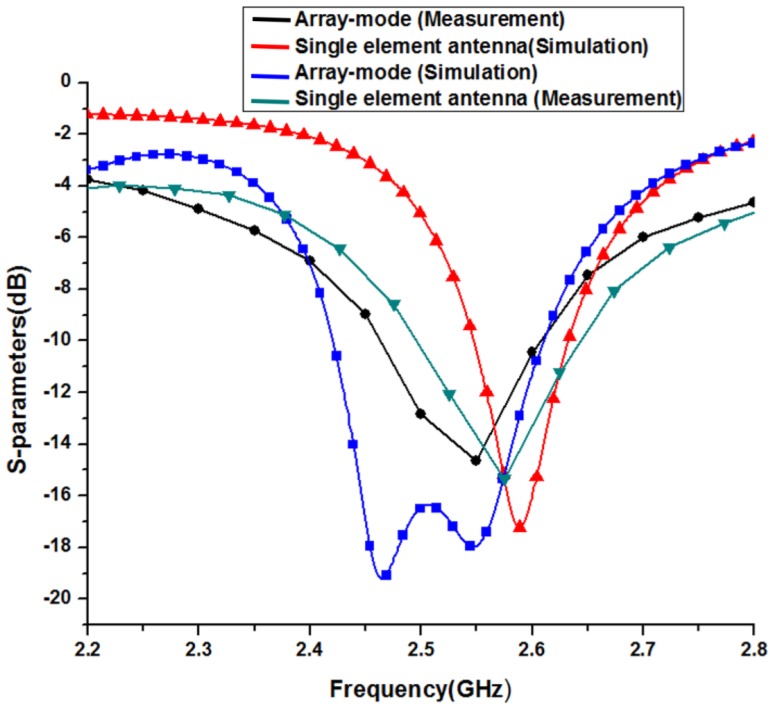
Simulated and measured S-parameters for the single antenna and array mode.

Copper tape was attached to the bottom side of the 3D-printed dielectric to serve as the antenna ground plane. Conductive silver epoxy (CW2400, Electronics Materials Co., Daejoo, Korea) was used to connect an SMA connector to the microstrip line. The volume resistivity of CW2400 is less than 0.001 Ω·cm; therefore, it has enough conductivity. The final antenna prototypes in the single antenna and array modes are shown in Figure 4. The reflection coefficient of the single antenna was measured by using an Anritsu vector network analyzer. The measured 10-dB impedance bandwidth was 2.5–2.65 GHz in the single antenna mode. Then, the second and third antenna elements were pulled out, and the reflection coefficient for the array mode was measured. In the array mode, the 10-dB impedance bandwidth was 2.48–2.62 GHz. The simulated and measured reflection coefficients for the single antenna element and array mode are shown in Figure 5.

**Figure 6:**
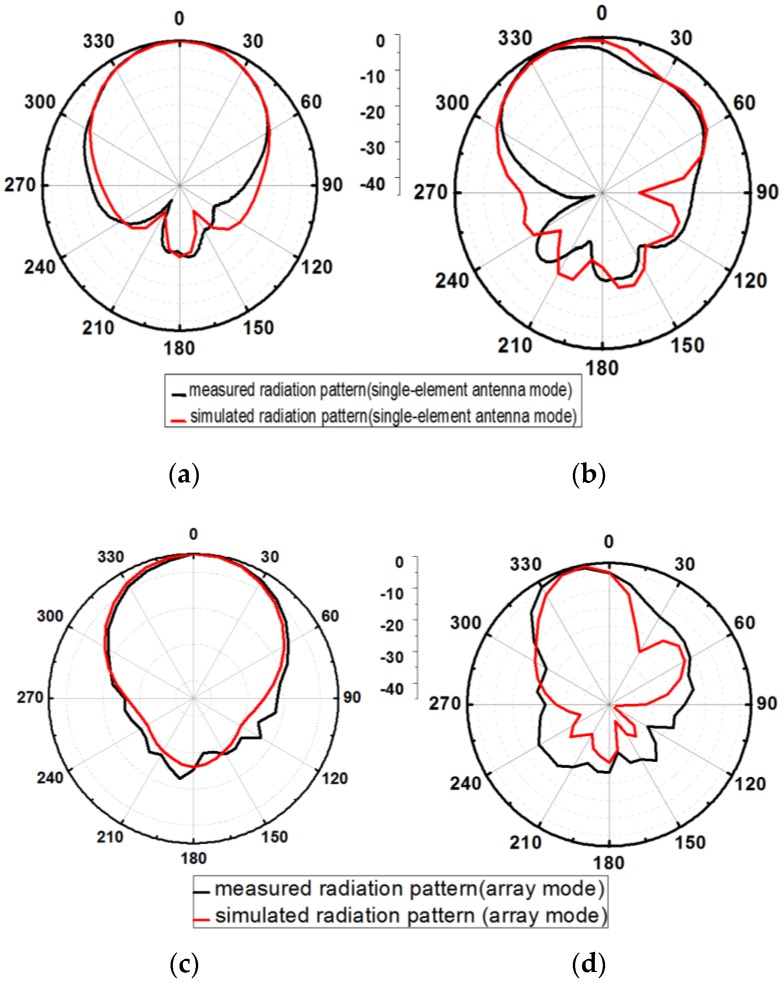
Simulated and measured normalized radiation pattern for (**a**) single-element antenna mode on the XZ plane (**b**) single-element antenna mode on the YZ plane. (**c**) Array mode on the XZ plane and (**d**) array mode on the YZ plane.

Both modes have almost the same resonance frequency. The radiation patterns of the single and array modes were measured in an anechoic chamber. In Figure 6a,b, the simulated and measured normalized radiation patterns are shown for the single-element antenna mode in the XZ and YZ planes. The measured gain of the single antenna is 5 dBi and 5.8 dBi in the XZ and YZ planes, respectively. In Figure 6c,d, the simulated and measured normalized radiation patterns are plotted for the array mode in the XZ and YZ planes. The measured gain of the antenna is 7.44 dBi and 7.63 dBi in the XZ and YZ planes, respectively. The slight difference between the simulated and measured return losses is due to the dielectric loss of the bonding film. Bonding film is employed between the inkjet printed paper and 3D printed PLA as well as between ground plane (copper tape) and PLA. In addition, the difference between the simulated and measured radiation patterns is due to the movable frames of the fabricated antenna.

## 4. Conclusions

A push/pull origami antenna that provides the flexibility to be operated both as a single antenna and as a 3 × 1 series array is proposed. The antenna has three elements with different frame sizes. In limited space, the frame of the third antenna element is pushed into the frame of the second antenna element, which is pushed under the first antenna element. In this mode, the antenna operates as a single element antenna. When the available space for the antenna is not limited and a higher gain is required, the proposed origami antenna can be transformed into a series array by pulling the second and third antenna elements. The proposed origami antenna was built using hybrid printing technology. This type of antenna can play an important role in remote-sensing applications.

## Figures and Tables

**Table 1:** Geometric parameters of the 3D-printed frames and array elements.

Parameter	Dimension (mm)	Parameter	Dimension (mm)
L	72	W	122
L_s1_	127	W_ms2_	8
W_s1_	120	L_p1_	50
L_s2_	90.2	W_p1_	60
W_s2_	122	L_i1_	14
H_s2_	6.5	W_p2_	80
L_s3_	77	L_p2_	46
W_s3_	120	L_i2_	6
H_s3_	5	L_p3_	46
L_ms1_	40	W_p3_	34
W_ms1_	15	G	0.2
L_ms2_	26	L_s_	14
L_ms3_	22	L_i1_	14
L_ms4_	22	L_i2_	6
